# Supplementary material for: Hsp70 chaperones, Ssa1 and Ssa2, limit poly(A) binding protein aggregation
Source: bioRxiv. 2025 Jan 20:2025.01.17.633617. Preprint. [Version 1] doi: 10.1101/2025.01.17.633617 (PMC11785122; doi:10.1101/2025.01.17.633617)
Supplement: Supplement 1 [file media-1.pdf]

SUPPLEMENTAL DATA

TITLE Hsp70 chaperones, Ssa1 and Ssa2, limit poly(A) binding protein aggregation

AFFILIATIONS Hannah E. Buchholz[1], Sean A. Martin[1], Jane E. Dorweiler[1], Claire M. Radtke[1], Adam S. Knier[1], Natalia B. Beans[1], Anita L. Manogaran[1]

1 Department of Biological Sciences, Marquette University, Milwaukee, WI, 53201-1881 USA

RUNNING HEAD Hsp70 limits Pab1 aggregation

ABBREVIATIONS Heat shock response (HSR), stress granule (SG), intrinsically disordered region (IDR), J-Domain proteins (JDP)

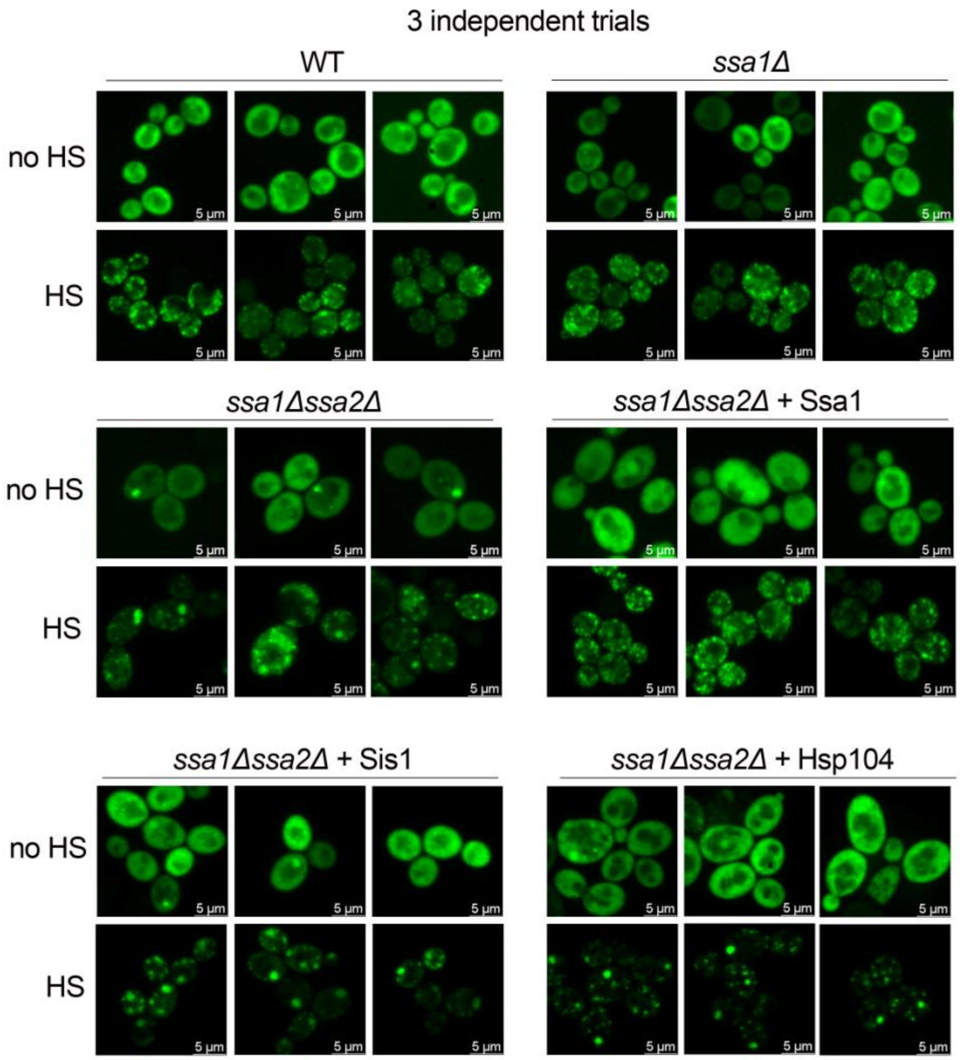

Supplemental figure 1. Additional representative images relevant to Figure 1A and B. Three independent images are shown of Pab1-GFP in the indicated strains, without (no HS) and with (HS) heat shock.

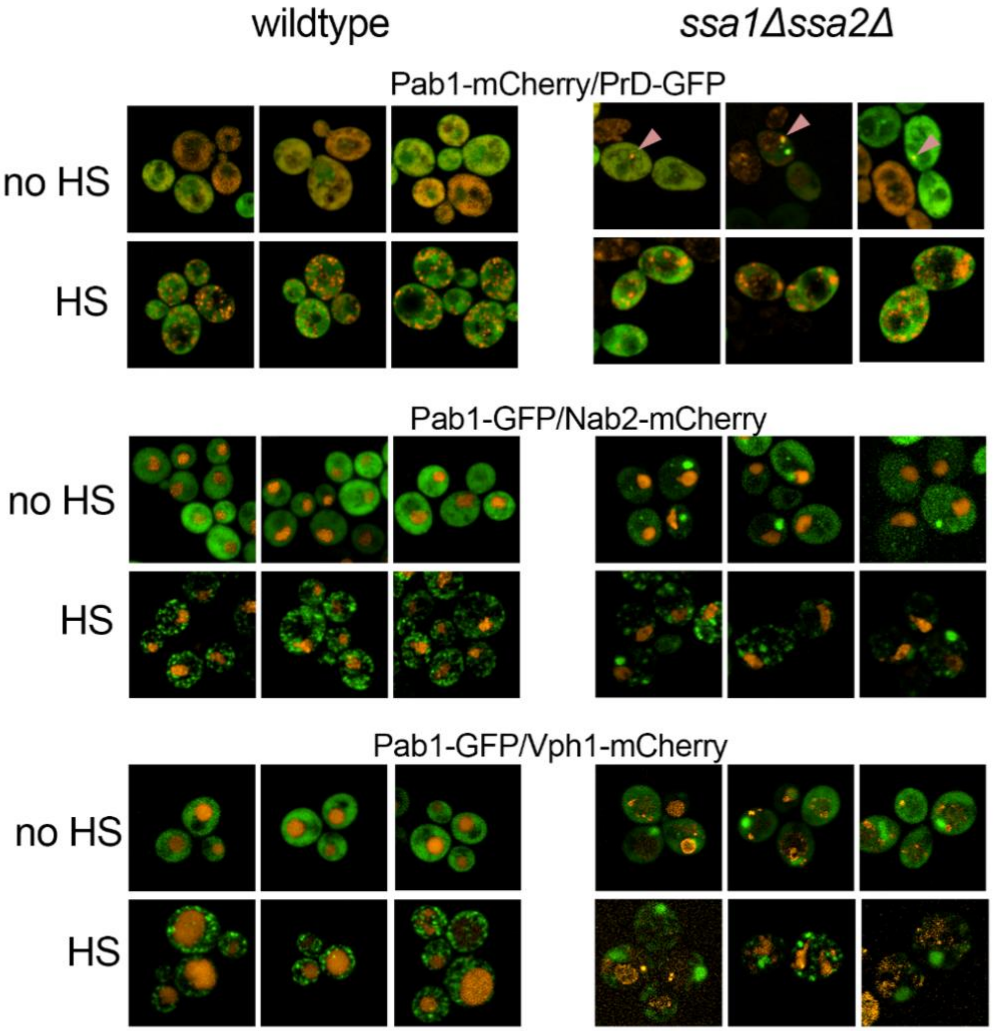

Supplemental figure 2. Additional merged representative images relevant to Figure 2. Three independent images are shown of Pab1-GFP in the indicated strains, without (no HS) and with (HS) heat shock. PrD-GFP is the prion domain of Sup35 fused to GFP, Nab2-mCherry localizes to the nucleus, and Vph1-mCherry localizes to the vacuole.

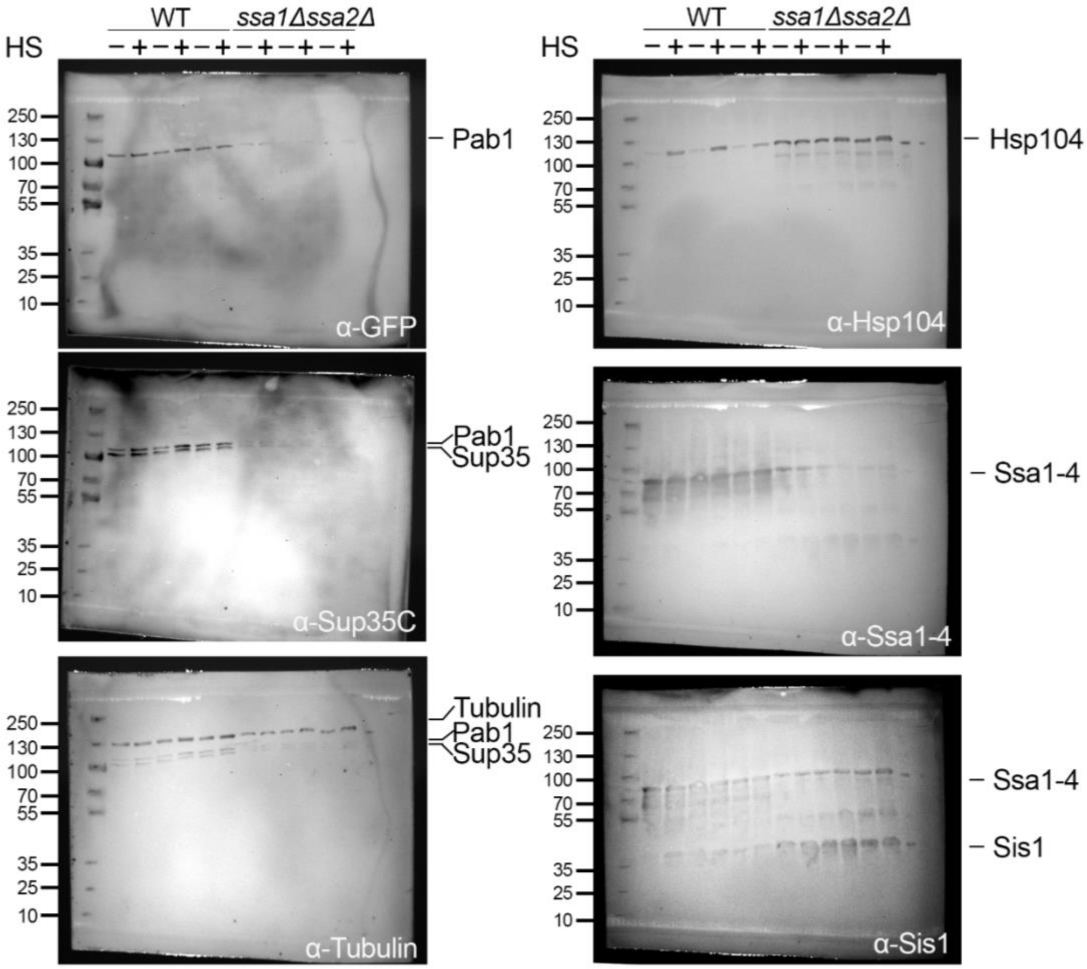

Supplemental figure 3. Raw Western blot gels relevant to Figure 3. Raw Western blot images with three samples of wildtype and *ssa1Δssa2Δ* without (-) and with (+) heat shock run on the same gel is shown. Molecular weight markers are shown on the left. Left. The same blot was incubated with antibodies for Pab1 (top), the C-terminus of Sup35 (middle), and Tubulin (bottom) in sequence using the same type of secondary antibody. Protein signals are indicated. Right. Blots from the left were also incubated with antibodies to

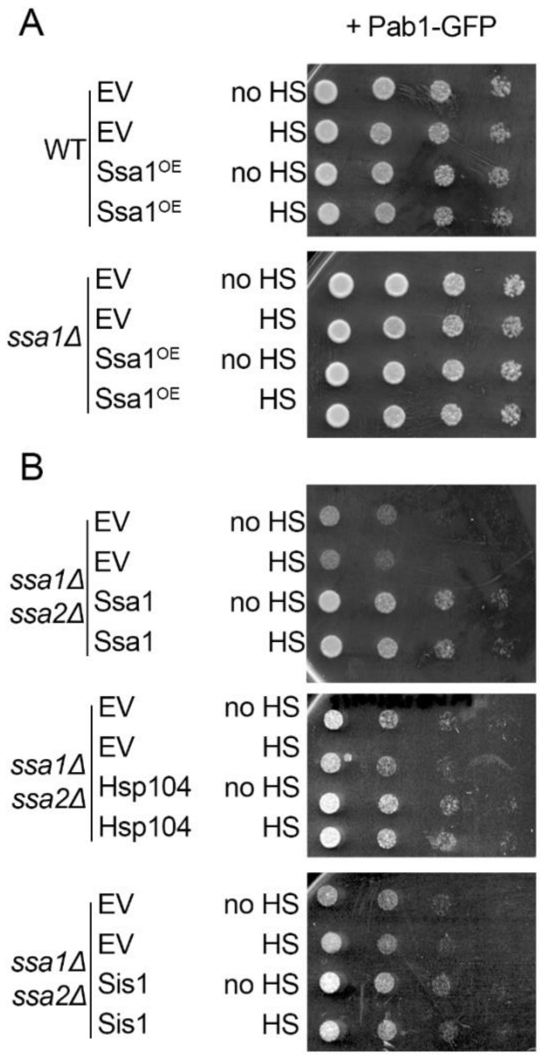

Hsp104 (top), Ssa1-4, (middle), and Sis1 (bottom) using a different secondary antibody. Protein signals are indicated.

Supplemental figure 4. *ssa1Δssa2Δ* strains exhibit a growth defect that is rescued by Ssa1 overexpression. A) Wildtype and *ssa1Δ* strains were transformed with Pab1-GFP and either a control plasmid (empty vector; EV) or GPD-Ssa1 overexpression plasmid (Ssa1). Cells were incubated at room temperature (no HS) or at 42°C for 45 minutes (HS). Strains were serially diluted five-fold, spotted on synthetic plasmid-selective media, and grown at 30°C for 2 days. B) Same as A, but in *ssa1Δssa2Δ* containing Pab1-GFP and either EV, GPD-Ssa1, HSE-Hsp104, or GPD-Sis1 overexpression plasmids.

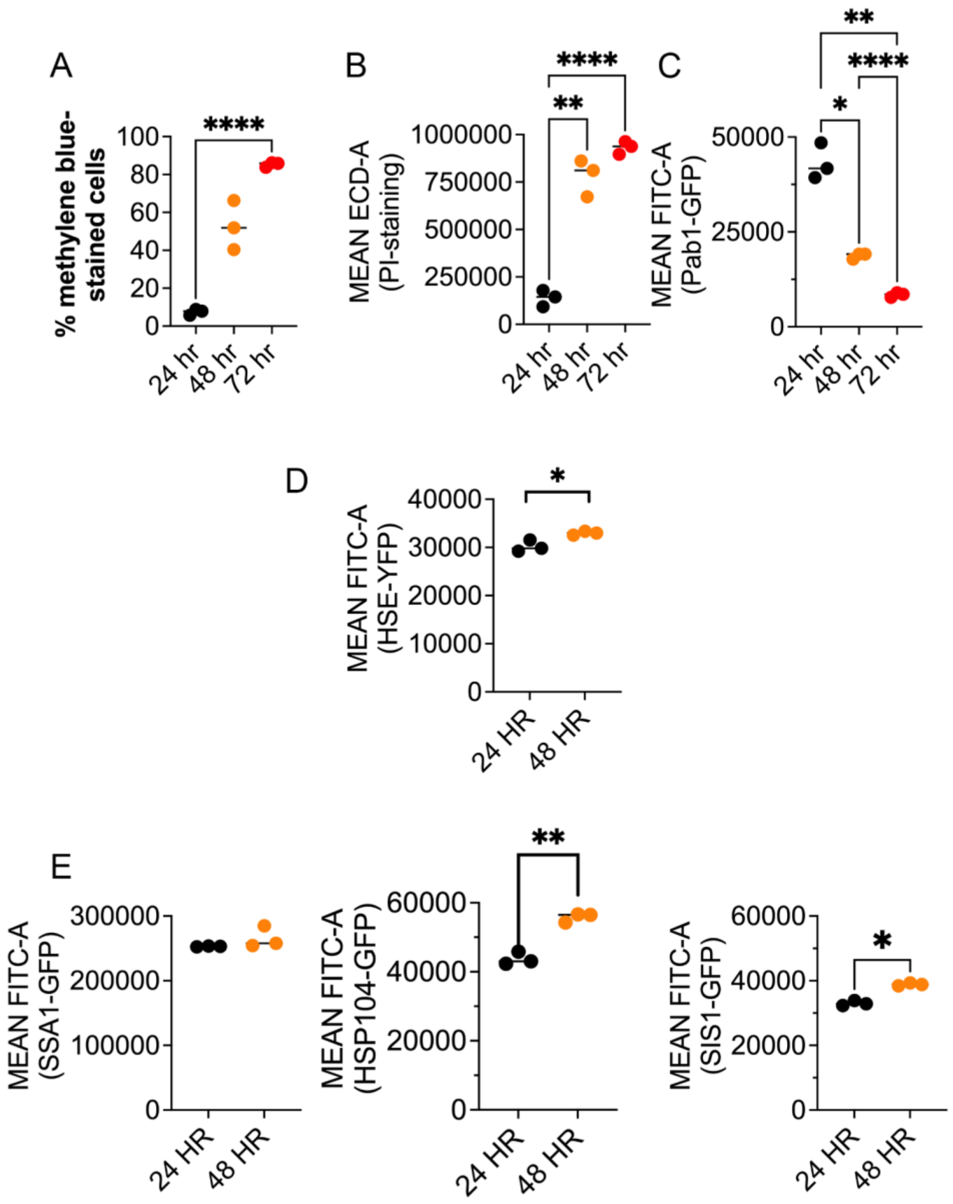

Supplemental figure 5. Pab1-GFP intensity and cell viability decreases, while HSR and chaperones increase in saturated cultures. A) Paired cultures were grown for the indicated time and stained with methylene blue to quantify the percentage of dead cells over time via brightfield microscopy. B and C) The same cells in A were also subjected to flow cytometry to quantify the amount of dead cells over time that were stained with propidium iodide (PI) using the ECD-A filter (B) and quantify the mean intensity of Pab1-GFP using the FITC-A filter (C). Means were compared by one way ANOVA (*p=0.0211; **p≤0.0098; ****p≤0.0001) D) Three independent cultures of a WT strain integrated with an HSE-YFP reporter were grown for 48 hours. YFP intensity was quantified through flow cytometry using the FITC-A filter and compared by paired t-test (*p=0.0397). E) Ssa1 (left),

Hsp104 (middle), and Sis1 (right) were endogenously tagged with GFP. Three independent cultures were grown for 48 hours and chaperone-GFP intensity was quantified through flow cytometry using the FITC-A filter and compared by paired t-test (*p=0.0121; **p=0.0075).

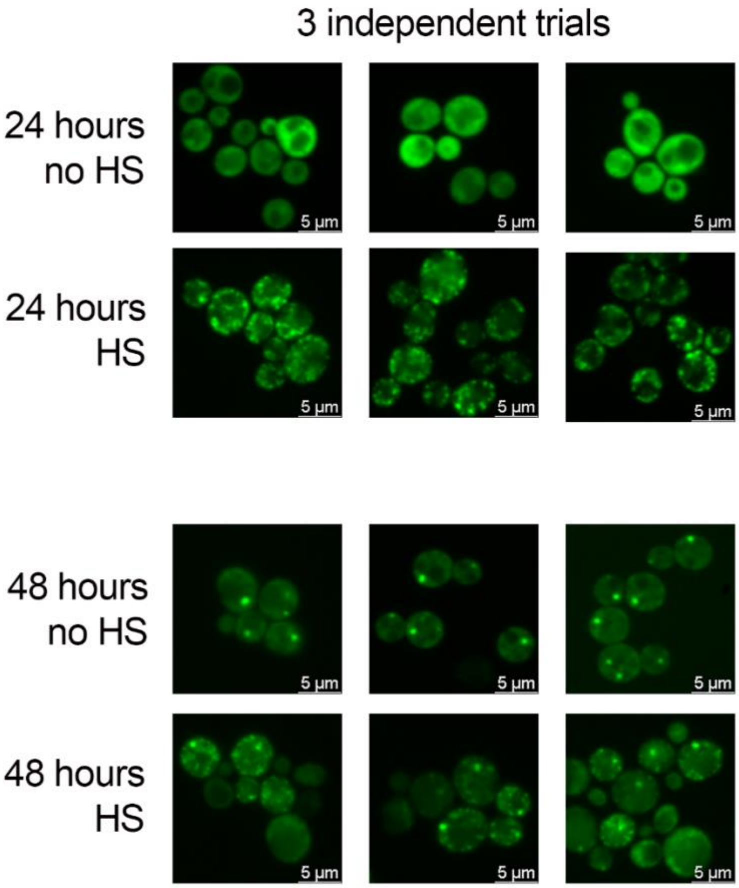

Supplemental figure 6. Additional representative images relevant to Figure 5.

Supplemental table 1. Yeast strains used in this study.

Strain #	Genotype	Name	Reference and figure
D230	Mat**a** ade1-14 ura3-52 leu2-3,112 trp1-289 his3-200	WT<br>74D-694	(Chernoff et al., 1995)<br>(All figures)
M675	Mat**a** ade1-14 ura3-52 leu2-3,112 trp1-289 his3-200 ssa1::HIS3 ssa2::NatR	*ssa1Δssa2Δ*<br>74D-694	This study.<br>Figures 1-4, SF1-4
M652	Mat**a** ade1-14 ura3-52 leu2-3,112 trp1-289 his3-200 ssa1::HIS3	*ssa1Δ*<br>74D-694	This study.<br>Figures 1, 4, SF1, SF4
M761	Mat**a** ade1-14 ura3-52 leu2-3,112 trp1-289 his3-200<br>Nab2-mCherry::TRP1	WT<br>74D-694<br>Nab2-mCherry	This study.<br>Figure 2
M757	Mat**a** ade1-14 ura3-52 leu2-3,112 trp1-289 his3-200 ssa1::HIS3 ssa2::NatR Nab2-mCherry::TRP1	*ssa1Δssa2Δ*<br>74D-694<br>Nab2-mCherry	This study.<br>Figure 2
M762	Mat**a** ade1-14 ura3-52 leu2-3,112 trp1-289 his3-200 Vph1-mCherry::TRP1	WT<br>74D-694<br>Vph1-mCherry	This study.<br>Figure 2
M763	Mat**a** ade1-14 ura3-52 leu2-3,112 trp1-289 his3-200 ssa1::HIS3 ssa2::NatR<br>Vph1-mCherry::TRP1	*ssa1Δssa2Δ*<br>74D-694<br>Vph1-mCherry	This study.<br>Figure 2

M703	Mat**a** ade1-14 ura3-52 leu2-3,112 trp1-289 his3-200 4xHSE-YFP::LEU2	WT<br>74D-694<br>4xHSE-YFP	This study.<br>Figure 3, SF5
M707	Mat**a** ade1-14 ura3-52 leu2-3,112 trp1-289 his3-200 ssa1::HIS3 ssa2::NatR 4xHSE-YFP::LEU2	*ssa1Δssa2Δ*<br>74D-694<br>4xHSE-YFP	This study.<br>Figure 3
M771	Mat**a** ade1-14 ura3-52 leu2-3,112 trp1-289 his3-200 ssa1::HIS3 4xHSE-YFP::LEU2	*ssa1Δ*<br>74D-694<br>4xHSE-YFP	This study.<br>Figure 3
M248	Matα ade1-14 his3-200 trp1-289 ura3-52 leu2-3,112 HSP104GFP::KANMX6	Hsp104-GFP<br>74D-694	Cured of prions<br>(Klaips et al., 2014)<br>SF5
M249	Mat**a** ade1-14 his3-200 trp1-289 ura3-52 leu2-3,112 SISGFP::KANMX6	Sis1-GFP<br>74D-694	Cured of prions<br>(Klaips et al., 2014)<br>SF5
M250	Mat**a** ade1-14 his3-200 trp1-289 ura3-52 leu2-3,112 SISGFP::KANMX6	Sis1-GFP<br>74D-694	Cured of prions<br>(Klaips et al., 2014)<br>SF5

Supplemental table 2. Plasmids used in this study.

Plasmid #	Description	Yeast Marker	Reference and figure
3284	Pab1-GFP	*URA3*	(Brengues and Parker, 2007) All figures
3285	Pab1-GFP	*TRP1*	(Brengues and Parker, 2007)Figures 1, 4, SF1, SF4
3272	Nab2-mCherry	*TRP1*	(Zhu et al., 2019)Figure 2, SF2
3273	Vph1-mCherry	*TRP1*	(Zhu et al., 2019)Figure 2, SF2
3029	pCup-Sup35PrD-GFP	*URA3*	(Zhou et al., 2001)Figure 2, SF2
3384	Pab1-mCherry	*TRP1*	This study. Figure 2, SF2
3357	4xHSE-YFP	*LEU2*	(Zheng et al., 2016)Figure 3, SF5
3110	pRS316 HSE-Hsp104	*URA3*	(Jackrel and Shorter, 2014)Figures 1, 4, SF1, SF4
3302	pAG415 GPD-Ssa1	*LEU2*	This study. Figures 1, 4, 5, SF1, SF4
3281	pAG414 GPD-Sis1	*TRP1*	This study. Figures 1, 4, SF1, SF4
3109	pRS316 HSE-EV	*URA3*	(Jackrel and Shorter, 2014)Figures 1, 4, SF1, SF4
3168	pRS315 HSE-EV	*LEU2*	(Jackrel and Shorter, 2014)Figures 1, 4, SF1, SF4

Supplemental table 3. Antibody and conditions used in this study.

Antibody	Dilution	Clonality	Vendor
GFP	1:5000	Monoclonal	Roche
Sup35C	1:10000	Monoclonal	Cocalico Biologicals
Ssa1-4	1:10000	Polyclonal	Elizabeth Craig
Hsp104	1:10000	Polyclonal	Enzo
Sis1	1:10000	Polyclonal	Elizabeth Craig
Tubulin	1:10000	Monoclonal	Invitrogen

REFERENCES

Brengues, M., and R. Parker. 2007. Accumulation of polyadenylated mRNA, Pab1p, eIF4E, and eIF4G with P-bodies in Saccharomyces cerevisiae. *Mol Biol Cell*. 18:2592-2602. PMC1924816

Chernoff, Y.O., S.L. Lindquist, B. Ono, S.G. Inge-Vechtomov, and S.W. Liebman. 1995. Role of the chaperone protein Hsp104 in propagation of the yeast prion-like factor [psi+]. *Science*. 268:880-884. PMID7754373

Jackrel, M.E., and J. Shorter. 2014. Potentiated Hsp104 variants suppress toxicity of diverse neurodegenerative disease-linked proteins. *Dis Model Mech*. 7:1175-1184. PMC4174528

Klaips, C.L., M.L. Hochstrasser, C.R. Langlois, and T.R. Serio. 2014. Spatial quality control bypasses cell-based limitations on proteostasis to promote prion curing. *eLife*. 3. PMC4270096

Zheng, X., J. Krakowiak, N. Patel, A. Beyzavi, J. Ezike, A.S. Khalil, and D. Pincus. 2016. Dynamic control of Hsf1 during heat shock by a chaperone switch and phosphorylation. *eLife*. 5. PMC5127643

Zhou, P., I.L. Derkatch, and S.W. Liebman. 2001. The relationship between visible intracellular aggregates that appear after overexpression of Sup35 and the yeast prion-like elements [PSI(+)] and [PIN(+)]. *Mol Microbiol*. 39:37-46. PMID11123686

Zhu, J., Z.T. Zhang, S.W. Tang, B.S. Zhao, H. Li, J.Z. Song, D. Li, and Z. Xie. 2019. A Validated Set of Fluorescent-Protein-Based Markers for Major Organelles in Yeast (Saccharomyces cerevisiae). *mBio*. 10. PMC6722415
